# Exploring differences between women and men in treatment-seeking patients with compulsive buying-shopping disorder

**DOI:** 10.1038/s41598-026-43027-4

**Published:** 2026-03-04

**Authors:** Bjarn-Ove Tetzlaff, Tanja Bogel, Tobias A. Thomas, Nora M. Laskowski, Astrid Müller

**Affiliations:** 1 Department of Psychosomatic Medicine and Psychotherapy, Hannover Medical School, Carl-Neuberg-Straße 1, 30625 Hannover, Germany; 2 Department of Psychiatry, Social Psychiatry and Psychotherapy, Hannover Medical School, Carl-Neuberg-Straße 1, 30625 Hannover, Germany; 3 University Clinic for Psychosomatic Medicine and Psychotherapy, Medical Faculty, Ruhr-University Bochum, Campus East-Westphalia, Virchowstraße 65, 32312 Lübbecke, Germany

**Keywords:** Compulsive buying-shopping disorder, Gender differences, Comorbid mental disorders, Buying/shopping preferences, Diseases, Health care, Medical research, Psychology, Psychology

## Abstract

Compulsive buying-shopping disorder (CBSD) is associated with emotional distress, reduced daily functioning, and frequent comorbid mental health conditions. In previous research, CBSD has traditionally been conceptualized as a predominantly female disorder, with most existing data derived from primarily female samples. This study examined gender differences in a treatment-seeking sample with CBSD, focusing on sociodemographic factors, mental comorbidities, buying/shopping preferences, symptom severity, and therapy participation. We conducted a retrospective cross-sectional analysis of 141 adults (73.8% women) diagnosed with CBSD at an outpatient clinic for behavioral addictions (2017–2025). Data included sociodemographic variables, ICD-10 comorbidities, buying/shopping environment, preferred consumer products, and group therapy attendance. Psychometric measures (Pathological Buying Screener, PBS; Generalized Anxiety Disorder Scale 7, GAD-7; Patient Health Questionnaire, PHQ-9) assessed symptoms of CBSD, anxiety, and depression. Group comparisons used Chi-square and Mann-Whitney U tests. Women more often compulsively purchased clothing, shoes, bags, cosmetics, and jewelry (all *p* ≤ .004, *V* = 0.18–0.36); men preferred electronics (*p* < .001, *V* = 0.32). Women showed slightly higher CBSD severity (PBS: Mdn = 56 vs. 52, *p* = .044, *r* = .17). Overall, no significant differences between women and men were found in comorbid mental disorders, neither in GAD-7/PHQ-9 scores (all *p* > .05) nor in clinical diagnoses (all *p* > .004 after Bonferroni correction). No group differences emerged for age, education, relationship status, or therapy participation (all *p* > .05). Gender differences in CBSD presentation—particularly regarding product preferences—emphasize the need for gender-sensitive perspectives to enhance treatment outcomes, while also highlighting that men and women differ only in specific aspects.

## Introduction

Compulsive buying-shopping disorder (CBSD) is characterized by an intense urge to engage in and excessive preoccupation with buying/shopping, and significantly impaired control over buying/shopping behavior^1,2^. Individuals frequently purchase consumer goods that are often neither needed nor used^2,3^. Despite a range of negative consequences—such as financial difficulties, family conflicts, and reduced quality of life^2–5^—individuals with CBSD often find themselves unable to stop. They also frequently suffer from comorbid mental disorders, including anxiety, depression, hoarding disorder, personality disorders, and impulse control disorders^1,2,6–9^. A 2016 meta-analysis estimated the prevalence of CBSD in the general population to be approximately 5%^10^. The increasing digitalization of consumer behavior and the shift towards more online buying/shopping may further exacerbate the phenomenon^11^. CBSD is listed as an example in the ICD-11 category *Other specified impulse control disorders* (6C7Y)^12^.

An early study by Schlosser and colleagues^16^ described the prototypical individual with CBSD as female. Previous research predominantly conceptualized CBSD as occurring more frequently among women^7^. In some research samples, exclusively women were recruited^13–16^. However, a recent literature review by Laskowski et al.^17^, which included 39 studies examining gender differences in CBSD prevalence estimates, revealed a heterogeneous set of findings. In 19 studies, men and women exhibited CBSD at comparable rates; another 19 studies showed higher rates than in men; and in one study, men were more frequently affected^17^. Only four studies used clinical samples^6,8,17,18^. Thus, it remains unclear whether women consistently have a higher risk of developing CBSD or suffer from higher CBSD severity and therefore seek more often treatment for CBSD than men^2,8^.

Previous findings have highlighted gender differences in the types of preferred items during buying/shopping episodes. Findings from a study conducted 20 years ago indicated that women were more inclined to compulsively purchase items with emotional impact, while men tended to favor functional products^19^. Studies have identified distinct preferences in the purchase of items, with women prefer clothing, jewelry, shoes, perfumes, and cosmetics, while men are more likely to buy electronic devices, cars, suits, and watches^20,21^. Furthermore, men tend to look for information when buying/shopping, while women are more concerned with gratification^22^. Differences in purchasing preferences may be essential, as the acquired items are crucial in conveying the relieving and rewarding effects of buying/shopping^2^. Dittmar argued that women associate shopping more strongly with emotions and that it concerns aspects of identity in women rather than in men^23^. She further described shopping to be more of a hedonistic leisure activity whereas for men, it would be more of a task that needs to be completed. Thus, men would strive for efficiency in shopping environments. Shopping might be of a deeper meaning for women and might also be an opportunity to express themselves and convey their identity. Importantly, Dittmar argued that these facts must be considered against their sociocultural background. Gender differences in buying behavior in general might occur as long as there are clear cultural norms and gender roles and as long as women are more restricted in expressing themselves and using emotion regulation strategies. This, according to Dittmar, also has to do with the fact that women tend to perform most of the housework and care work^23^. Given that this explanation was suggested twenty years ago and gender roles are transforming in many societies it remains a question for further research whether it would be still valid today. It also needs to be critically mentioned that although more and more research has been devoted to the field of CBSD in the past years, empirical and etiological psychological and biological studies on gender differences in compulsive buying still lack.

Further gender differences have been identified both in psychiatric comorbidities and in underlying psychological mechanisms associated with CBSD. With regard to psychiatric comorbidities, a Brazilian clinical sample showed that men with CBSD had higher rates of sex addiction and intermittent explosive disorder than women^20^. In the same study, however, no specific mental comorbidities were found to be more prevalent among women with CBSD. Beyond mental diagnoses, gender differences have also been reported with respect to psychological mechanisms. In non-clinical samples, CBSD in women has more frequently been associated with deficits in self-regulation, higher levels of self-uncertainty, and problems related to self-esteem^8,24,25^.

While various theoretical models have been proposed to explain CBSD, their consideration of gender is inconsistent. Some frameworks identify gender as a relevant antecedent or predictor—such as Workman and Paper’s model^26^, which links CBSD to gender, or the online shopping addiction model by Rose and Dhandayudham^27^, which includes female gender among its predictors. Other models, such as the cognitive-behavioral framework by Kellett and Bolton^28^, or the integrative model by Brand and Müller^29^, which applies core aspects of the Interaction of Person-Affect-Cognition-Execution (I-PACE) model of behavioral addictions^30,31^ and the components model of addiction to CBSD, do not incorporate gender at all. Similarly, a theoretical synthesis presented by Totzke et al.^32^, which integrates existing models of behavioral addictions and dual-process theories of decision-making, does not consider gender as a relevant factor.

Several key gaps remain in the CBSD literature. While some studies have already explored gender differences in CBSD in non-clinical samples^25,33^, few have focused specifically on treatment-seeking samples^8,20^. Men remain underrepresented, and their product preferences, symptom profiles, and treatment needs are not well understood. Few studies have examined gender differences across multiple domains, e.g. shopping behavior, comorbidities, and therapy engagement.

The present study aimed to contribute to the existing literature by examining whether women and men seeking treatment for CBSD differ in demographic characteristics, symptom severity, preferred shopping environment (i.e., offline and/or online) and consumer goods, mental comorbidities, and engagement in psychotherapy. For clinical and sociodemographic variables, an exploratory approach was adopted, as the literature remained inconclusive.

In contrast, for buying/shopping item preferences, we hypothesized that the above-mentioned gender-stereotypical product preferences would largely persist^19^. At the same time, given a potential shift in product preferences over time and a dissolution of traditional gender-specific consumer patterns^34^, societal changes and increasing flexibility in gender roles, we explored whether the strength of these differences differs from those reported in earlier studies.

## Method

### Procedure

The present retrospective cross-sectional study is based on data collected between June 2017 and February 2025. The sample consists of German-speaking patients who presented to the Outpatient Clinics for Behavioral Addictions at Hannover Medical School and Klinikum Nürnberg during this period for routine clinical assessment and treatment. Patients were not recruited via a study call but sought help on their own initiative, completed standardized questionnaires as part of the regular intake assessment, and subsequently underwent a clinical diagnostic interview in which ICD-10 diagnoses were established and communicated to the patients. All participants actively sought help, and only adults who met the proposed diagnostic criteria for compulsive buying^3^ were included in the study. Thus, the sample comprises individuals with clinically relevant and more severe forms of CBSD, which may be associated with very high levels of distress and impairment, as reflected by PBS scores substantially above the clinical cut-off^35,36^ and higher mean symptom severity compared to mixed community-based samples^37^. Due to the small number of participants, patients who stated that they had a non-binary gender (*n* = 2) were excluded. Furthermore, individuals with learning or developmental disorders, current or past manic episodes, or acute suicidality were excluded during recruitment.

As part of the data collection, we assessed sociodemographic information and details regarding the patients’ buying/shopping behavior. Additionally, participants completed a set of questionnaires. These questionnaires were completed before the initial consultation, prior to any CBSD-related treatment. After that, a routine clinical interview was conducted by supervised licensed psychotherapists with extensive clinical experience and specific expertise in psychopathology, particularly in the field of CBSD. The clinical interview was used to verify whether the diagnostic criteria proposed by McElroy et al.^3^ were met, to detect the duration of CBSD and to assess comorbid mental disorders according to the ICD-10^38^. We also examined how many participants started group therapy—consisting of 12 to 13 weekly CBT sessions with 6 to 8 patients per group— and continued for at least three sessions after the initial consultation.

Written informed consent was obtained from all participants prior to data collection, including consent for the use of their data for future research purposes and for the publication of the corresponding results. The data were pseudonymized, and the study was approved by the Ethics Committee of Hannover Medical School (No. 11366_BO_S_2024). All procedures were conducted in accordance with institutional and national ethical standards and the 1975 Helsinki Declaration. Subsamples (*n* = 81; *n* = 128) of the data used in the present study have previously been analyzed by our research group in the context of further research questions^1,9^.

### Materials

The participants provided socio-demographic information and details of their purchasing preferences in a paper survey. Firstly, they stated their age, years of school education, and partnership status. Then, they reported whether they preferred to buy/shop online, offline, or mixed online and offline. We also evaluated participants’ preferred items, enabling them to select the following options: bags, books, CDs, clothing, cosmetics, household goods, jewelry, shoes, food, small electronic devices, large electronic devices, and sports equipment.

We used the Pathological Buying Screener (PBS;^36^ to assess symptoms of CBSD. The self-rating tool consists of 13 items assessing for instance negative consequences, concealment tendencies, and reduced control over buying/shopping behavior in the last six months (e.g., “How often did you buy more things than you need?“). Responses are given on a 5-point Likert scale (1 = “never”, 5 = “very often”), with higher total scores indicating greater severity of CBSD. One hundred thirty-nine participants (98.6%) exceeded the cut-off score of ≥ 29, which Müller et al.^36^ proposed as an indicator of CBSD in clinical samples^35^. The internal consistency of the PBS was good in the present sample, with Cronbach’s alpha = 0.83.

We used the German version of the Generalized Anxiety Disorder Scale 7 (GAD-7;^39^ to assess symptoms of generalized anxiety disorder (e.g., “worrying too much about different things”). The scale provides seven items and measures the frequency of anxiety symptoms experienced over the past two weeks using a four-point Likert scale (0 = “not at all” to 3 = “nearly every day”). The scale’s internal consistency was good, Cronbach’s alpha = 0.84.

To assess the severity of depression, the German version of the depression module of the Patient Health Questionnaire (PHQ-9;^40^ was used. This module consists of 9 items, which measure the frequency of depressive symptoms over the past two weeks (e.g., “little interest or pleasure in doing things”) on a four-point Likert scale (0 = “not at all” to 3 = “nearly every day”). The internal consistency was good, with Cronbach’s alpha = 0.88.

### Participants

The total sample comprised *N* = 141 patients with a mean age of 41.41 years (*SD* = 13.36, Min = 18, Max = 70). One hundred and four (73.8%) patients were women, and 37 (26.2%) were men. Regarding education, 93 patients (67.4%) stated that they had attended school for more than 12 years, while 45 (32.6%) reported receiving 12 years of schooling at most. Three patients did not provide information on their education. In terms of relationship status, 76 (56.7%) participants were in a relationship, 58 (43.3%) were not, and 7 did not specify.

For the statistical evaluation, we summarized the comorbid mental disorders into the following groups: Thirty-three (23.4%) were diagnosed with hoarding disorder (ICD-10: F42.8). Depression (ICD-10: F32.x, F33.x, F34.x, F38.1) was present in 103 patients (73%). Anxiety disorders (ICD-10: F40.x, F41.x) were diagnosed in 11 patients (7.8%), with generalized anxiety disorder being the most frequent subtype (*n* = 5), followed by specific phobias (*n* = 2), agoraphobia with panic disorder (*n* = 2), mixed anxiety and depressive disorder (*n* = 1), and agoraphobia without panic disorder (*n* = 1). Post-traumatic stress disorder (PTSD; ICD-10: F43.1) was found in 28 patients (19.9%), and obsessive-compulsive disorder (OCD; ICD-10: F42.0-F42.2) in 2 patients (1.4%). Adjustment disorders (ICD-10: F43.2, F43.8, F43.9) were diagnosed in 3 patients (2.1%). Seventeen patients (12.1%) had a somatoform disorder (ICD-10: F44, F45, F48), while eating disorders (ICD-10: F50) were diagnosed in 28 patients (19.9%). Schizophrenia (ICD-10: F20, F22, F25) was present in 5 patients (3.5%). Personality disorders (ICD-10: F60, F61) were found in 28 patients (19.9%). Attention deficit hyperactivity disorder (ADHD; ICD-10: F90.0) and substance use disorder (ICD-10: F10.x-F19.x) were each diagnosed in 9 patients (6.4%).

We conducted a post-hoc power analysis using G*Power (version 3.1;^41^ for the Mann-Whitney U test, which represented the most statistically demanding analysis conducted in this study in terms of sample size requirements. Assuming a medium effect size (d = 0.50), an alpha level of 0.05, and two independent groups (women: *n* = 104; men: *n* = 37), the achieved power (1 – β) was 0.86. This suggests that the study was sufficiently powered to detect medium-sized group differences in CBSD severity and related outcomes.

### Statistical analysis

The data analysis was performed using IBM SPSS Statistics (version 29.0.1). The Chi-square test was used to examine differences in categorical variables including comorbid mental disorders, preferred shopping environment, buying/shopping item preferences, partnership status, and years of schooling. If the expected cell frequency was below 5, Fisher’s exact test was applied instead. Differences in metrically scaled variables, such as PBS scores, duration of CBSD, and age were analyzed using the Mann-Whitney U test. Since the Shapiro-Wilk test indicated that the assumption of normal distribution was not met in all groups, a non-parametric test was chosen. Effect sizes were calculated using Cramér’s *V* for chi-square tests and Bravais-Pearson’s *r* for Mann-Whitney *U* tests, following Cohen’s recommendations^42^ for interpretation (*V* = 0.10 small effect, *V* = 0.20 moderate effect, *V* = 0.30 large effect; *r* = .10 small effect, *r* = .30 moderate effect, *r* = .50 large effect).

Due to limited sample size and the distributional characteristics of the data, multivariate analyses were not feasible. Therefore, to control for inflated Type I error resulting from multiple univariate comparisons, a Bonferroni correction was applied.

Cases with missing data were excluded listwise only from analyses involving the respective variables. The exact sample size (n) for each analysis is reported accordingly for transparency.

## Results

We examined differences between women and men in clinical characteristics, comorbid disorders, therapy participation, and shopping-related behaviors among patients with CBSD. Group differences were found in symptom severity, psychiatric comorbidities, and item preferences, whereas no significant differences emerged in treatment engagement or symptom duration.

Our analyses revealed no significant differences between women and men in terms of age (*U* = 1600.50, *p* = .149, *n* = 140), years of schooling (*χ²*(1) = 0.63, *p* = .428, *n* = 138), or partnership status (*χ²*(1) < 0.01, *p* = .995, *n* = 134).

The results of the group comparisons for all diagnosed comorbid disorders are reported in Table 1. Depression was significantly more prevalent among women with CBSD compared to men with CBSD. Additionally, eating disorders were significantly more common in women than in men. A more detailed analysis revealed that binge-eating disorder was the most frequently diagnosed eating disorder in the sample and was significantly more prevalent among women than men. The effect sizes for differences between women and men in comorbid disorders ranged from small to moderate^42^, indicating that the observed group differences are of low to medium practical relevance^42^. Moreover, these differences did not remain statistically significant after Bonferroni correction (*p* > .004), further limiting their clinical relevance. The prevalence of all other comorbid disorders did not differ significantly between groups.

**Table 1:** Gender differences in patients with CBSD: Comorbid mental disorders.

Variable	Group		Test statistic	*p*	Effect size
	Men	Women			
Hoarding disorder (*N* = 141) Yes No	*n* = 37 12 (32.4%) 25 (67.6%)	*n* = 104 21 (20.2%) 83 (79.8%)	*χ²*(1) = 2.28	0.131	*V* = 0.13
Depression (*N* = 141) Yes No	*n* = 37 22 (59.5%) 15 (40.5%)	*n* = 104 81 (77.9%) 23 (22.1%)	*χ²*(1) = 4.70	0.030^*,a^	*V* = 0.18
Anxiety (*N* = 141) Yes No	*n* = 37 1 (2.7%) 36 (97.3%)	*n* = 104 10 (9.6%) 94 (90.4%)	Fisher’s exact test	0.178	*V* = 0.11
Generalized anxiety disorder (*N* = 141) Yes No	*n* = 37 0 (0%) 37 (100%)	*n* = 104 5 (4.8%) 99 (95.2%)	Fisher’s exact test	0.176	*V* = 0.11
PTSD (*N* = 141) Yes No	*n* = 37 5 (13.5%) 32 (86.5%)	*n* = 104 23 (22.1%) 81 (77.9%)	*χ²*(1) = 1.27	0.260	*V* = 0.10
OCD (*N* = 141) Yes No	*n* = 37 0 (0%) 37 (100%)	*n* = 104 2 (1.9%) 102 (98.1%)	Fisher’s exact test	0.396	*V* = 0.07
Adjustment disorder (*N* = 141) Yes No	*n* = 37 1 (2.7%) 36 (97.3%)	*n* = 104 2 (1.9%) 102 (98.1%)	Fisher’s exact test	1.00	*V* = 0.02
Somatic symptom disorder (*N* = 141) Yes No	*n* = 37 3 (8.1%) 34 (91.9%)	*n* = 104 14 (13.5%) 90 (86.5%)	Fisher’s exact test	0.559	*V* = 0.07
Eating disorders (*N* = 141) Yes No	*n* = 37 2 (5.4%) 35 (94.6%)	*n* = 104 26 (25%) 78 (75%)	Fisher’s exact test	0.010^*,a^	*V* = 0.22
Binge eating disorder (*N* = 141) Yes No	*n* = 37 2 (5.4%) 35 (94.6%)	*n* = 104 21 (20.2%) 83 (79.8%)	Fisher’s exact test	0.039^*,a^	*V* = 0.18
Substance use disorder (*N* = 141) Yes No	*n* = 37 1 (2.7%) 36 (97.3%)	*n* = 104 8 (7.7%) 96 (92.3%)	Fisher’s exact test	0.445	*V* = 0.09
Schizophrenia (*N* = 141) Yes No	*n* = 37 1 (2.7%) 36 (97.3%)	*n* = 104 4 (3.8%) 100 (96.2%)	Fisher’s exact test	1.00	*V* = 0.03
Personality disorder (*N* = 141) Yes No	*n* = 37 8 (21.6%) 29 (78.4%)	*n* = 104 20 (19.2%) 84 (80.8%)	*χ²*(1) = 1.00	0.754	*V* = 0.03
ADHD (*N* = 141) Yes No	*n* = 37 3 (8.1%) 34 (91.9%)	*n* = 104 6 (5.8%) 98 (94.2%)	Fisher’s exact test	0.698	*V* = 0.04

Effect size assessed with Cramér’s *V* for χ²-test and Fisher’s exact test. *p ≤ .05; ^a^ *p* > .004 (significance level after Bonferroni correction). PTSD = Post-traumatic stress disorder. OCD = Obsessive-compulsive disorder. ADHD = Attention deficit hyperactivity disorder. Binge eating disorder is included in eating disorders.

For an overview of the results concerning buying/shopping preferences, see Table 2. We found no significant group differences regarding the preferred shopping environment. Concerning the preferred buying/shopping items, women significantly more frequently chose bags, clothing, cosmetics, household goods, jewelry, shoes, and food than men. Men, on the other hand, significantly more frequently preferred large and small electronic devices than women. The effect sizes were moderate to large, suggesting that the preferences between women and men are of considerable practical importance^42^. After Bonferroni correction, the differences for household goods and food did not remain statistically significant (*p* > .004). Preferences for books, CDs, and sports equipment did not differ significantly between women and men.

**Table 2:** Gender differences in patients with CBSD: Buying/shopping preferences.

Variable	Group		Test statistic	*p*	Effect size
	Men	Women			
Buying/Shopping environment (*N* = 125) Online Offline Online and offline	*n* = 34 21 (61.7%) 1 (2.9%) 12 (35.4%)	*n* = 91 50 (54.9%) 10 (11.0%) 31 (34.1%)	Fisher’s exact test	0.607	*V* = 0.14
Product categories					
Bags (*N* = 126) Yes No	*n* = 34 6 (17.6%) 28 (82.4%)	*n* = 92 45 (48.9%) 47 (51.1%)	*χ²*(1) = 10.07	0.002**	*V* = 0.28
Books (*N* = 126) Yes No	*n* = 34 9 (26.5%) 25 (73.5%)	*n* = 92 31 (33.7%) 61 (66.3%)	*χ²*(1) = 0.60	0.439	*V* = 0.07
CDs (*N* = 126) Yes No	*n* = 34 6 (17.6%) 28 (82.4%)	*n* = 92 11 (12.0%) 81 (88.0%)	*χ²*(1) = 0.69	0.407	*V* = 0.07
Clothing (*N* = 126) Yes No	*n* = 34 21 (61.8%) 13 (38.2%)	*n* = 92 89 (96.7%) 3 (3.3%)	Fisher’s exact test	< 0.001**	*V* = 0.47
Cosmetics (*N* = 126) Yes No	*n* = 34 6 (17.6%) 28 (82.4%)	*n* = 92 53 (59.6%) 39 (40.4%)	*χ²*(1) = 15.92	< 0.001**	*V* = 0.36
Household goods (*N* = 125) Yes No	*n* = 34 7 (20.6%) 27 (79.4%)	*n* = 91 41 (45.1%) 50 (54.9%)	*χ²*(2) = 6.67	0.036^*,a^	*V* = 0.23
Jewelry (*N* = 126) Yes No	*n* = 34 6 (17.6%) 28 (82.4%)	*n* = 92 42 (45.7%) 50 (54.3%)	*χ²*(1) = 8.25	0.004**	*V* = 0.26
Shoes (*N* = 126) Yes No	*n* = 34 15 (44.1%) 19 (55.9%)	*n* = 92 75 (81.5%) 17 (18.5%)	*χ²*(1) = 17.02	< 0.001**	*V* = 0.37
Food (*N* = 125) Yes No	*n* = 33 8 (24.2%) 25 (75.8%)	*n* = 92 41 (44.6%) 51 (55.4%)	*χ²*(1) = 4.21	0.040^*,a^	*V* = 0.18
Small electronic devices (*N* = 126) Yes No	*n* = 34 18 (52.9%) 16 (47.1%)	*n* = 92 19 (20.7%) 73 (79.3%)	*χ²*(1) = 12.48	< 0.001**	*V* = 0.32
Large electronic devices (*N* = 126) Yes No	*n* = 34 9 (26.5%) 25 (73.5%)	*n* = 92 4 (4.3%) 88 (95.7%)	Fisher’s exact test	< 0.001**	*V* = 0.32
Sports equipment (*N* = 126) Yes No	*n* = 34 8 (23.5%) 26 (76.5%)	*n* = 92 17 (18.5%) 75 (81.5%)	*χ²*(1) = 0.40	0.528	*V* = 0.06

Effect size assessed with Cramér’s *V* for χ²-test. *p ≤ .05; **p ≤ .01; ^a^*p* > .004 (significance level after Bonferroni correction).

Men (70.3%) and women (61.5%) did not differ significantly in therapy participation (*χ²*(1) = 0.90, *p* = .342, *n* = 141). Women (Mdn = 56) showed significantly higher PBS scores than men (Mdn = 52), *U* = 1465, *p* = .044, *r* = .17, indicating a small effect, and thus only a small difference in CBSD symptom severity. The duration of CBSD in years did not significantly differ between the groups (Mdn = 13 for women, Mdn = 7 for men, *U* = 208.50, *p* = .103). Similarly, there were no significant gender differences in GAD-7 scores (Mdn = 15 for women, Mdn = 14 for men, *U* = 981.50, *p* = .176) or PHQ-9 depression module scores (Mdn = 14 for both groups, *U* = 1542.50, *p* = .387).

## Discussion

The present study aimed to examine differences between women and men in a treatment-seeking sample of individuals with compulsive buying-shopping disorder (CBSD). Overall, our results indicate that women and men with CBSD are largely comparable with regard to demographic characteristics, overall symptom severity, comorbid mental disorders, and treatment engagement.

### Product preferences

The most robust and clinically meaningful differences were found in buying/shopping item preferences, where moderate to large effects emerged. This finding mirrors previous findings^19–21^, indicating that stereotypical product preferences persisted: in line with our hypothesis, women reported shopping/buying items such as clothes, shoes, cosmetics, and food more frequently, while men reported greater involvement with electronics. The effect sizes suggested that these preferences are of practical relevance. These patterns remained stable despite the increased accessibility of a wide range of products through online platforms, suggesting that consumer preferences of women and men are robust and may be shaped by enduring social norms or identity-related motivations. Additionally, identity-related motivations, such as self-expression or social comparison, might further drive these gender-specific shopping patterns, as individuals align their purchasing behavior with culturally reinforced gender expectations. These findings also raise the question of whether the function of the purchased items differs by gender.

### Clinical severity and treatment engagement

While women in our sample reported slightly higher CBSD symptom severity (as measured by the PBS) compared to men, this difference was accompanied by a small effect size, indicating that the clinical significance of this finding is limited. Moreover, there were no differences between women and men in treatment participation, particularly in group therapy attendance. This finding suggests that once individuals have sought help, women and men with CBSD appear similarly burdened and comparably willing to engage in treatment. The higher proportion of women in our sample may reflect a general greater willingness to initiate help-seeking behavior, as previously noted in psychotherapy research^43^. The question of whether more women than men are affected by CBSD can currently only be determined assessing a representative population by using a diagnostic interview^17^. However, our findings suggest that the extent of impairment, the presence of psychiatric comorbidities, and the motivation to engage in therapy are not substantially different between men and women once they enter the healthcare system. This raises important theoretical considerations regarding the role of gender in CBSD. While prior research has often portrayed CBSD as a predominantly female disorder, our findings suggest that gender may not be a decisive risk factor for developing clinically relevant CBSD. Instead, other factors—such as materialistic value orientation, difficulties in emotion regulation, or self-esteem problems^24,25,33^—may be more central to the etiology of CBSD, potentially interacting with gender but not determined by it. These alternative risk factors may be equally distributed across women and men or exert their effects independently of gender, thereby calling into question the conceptualization of CBSD as a gender-specific disorder. Notably, no comprehensive theoretical model currently exists that systematically incorporates gender as a central determinant in the development or maintenance of CBSD. This aspect highlights a significant gap in our understanding: while gender-specific differences in product preferences may result from gender-specific buying/shopping motives, there is currently no unified theoretical account of how gender influences the development, progression, maintenance, or treatment response of CBSD. Findings in the field of behavioral addictions, which highlight not only gender-specific prevalence rates but also gender-specific psychological and neurobiological mechanisms^8,20^, underscore the need to consider gender more systematically in theoretical models of behavioral addictions, including CBSD. Future research should thus not only continue to explore gender differences descriptively but also clarify whether gender functions as a causal risk factor, a moderating variable, or a correlate of other underlying mechanisms. Integrating gender more systematically into theoretical frameworks—whether as a socialization factor, identity-relevant construct, or moderator of psychological vulnerabilities—may provide a more nuanced understanding of CBSD and inform gender-sensitive prevention and treatment approaches.

### Comorbid mental disorders

The overall high prevalence of comorbid mental disorders in both genders underscores the frequent association of CBSD with broader mental health issues. The prevalence rates observed in our total sample were, in comparison to the population-based German data reported by Jacobi and colleagues^44^, notably higher for depression, PTSD, eating disorders, and somatoform disorders (Jacobi et al.^44^: 9.3%, 2.3%, 0.9%, and 3.5%; our sample: 73%, 19.9%, 19.9%, 12.1%). We also found a higher prevalence of hoarding disorder than reported for representative samples (2.5%^45^, . These results are partly consistent with the findings of de Mattos et al.^20^, who also reported increased prevalence of PTSD, depression, and eating disorders. In our sample, women were more frequently diagnosed with depression and eating disorders, particularly binge-eating disorder^46^. These findings align with epidemiological data indicating a higher prevalence of both disorders among women^44^ and are further supported by our data, which show that women more often report a preference for food-related products in their buying/shopping behavior compared to men. These findings may also reflect gender-specific emotion regulation strategies in buying/shopping behavior. Research suggests that women are more likely to engage in internalizing coping styles, such as rumination and emotion-focused regulation^47^, which can manifest in maladaptive behaviors aimed at short-term mood enhancement^48^. Compulsive buying/shopping, as well as binge eating, may serve as a way to regulate negative affect temporarily^49,50^. In this sense, the comorbidity between CBSD and eating disorders^51,52^ in women may point to a shared mechanism of maladaptive coping, e.g., during a depressive episode. However, it should be noted that the slightly higher frequency of depression in women was observed only with regard to clinical diagnoses and not in PHQ-9 scores, and that the effect size was small, thereby limiting the clinical relevance of this finding. Moreover, the differences in the prevalence of depressive and eating disorders, as well as the difference in the preference for food, did not remain statistically significant after correction for multiple testing, further limiting the clinical relevance of these results. Regarding other comorbid mental disorders, men and women with CBSD appear to represent a relatively homogeneous group.

### Limitations

Some limitations of the present study should be noted. First, the study relied on a binary gender classification, which does not account for non-binary or gender-diverse individuals. This is particularly important given that individuals outside of binary gender categories may face unique risk factors for mental disorders, including higher rates of discrimination, social exclusion, and identity-related stress. Future research should address the experiences of individuals across a broader gender spectrum. Second, the selection of product categories was not guided by a systematic or theoretical framework, and qualitative data, including shopping motives, on the personal meaning of specific purchases were not evaluated. Third, although the diagnostic interview used to assess CBSD was based on proposed CBSD criteria^3^ and conducted by experienced clinicians, the lack of a structured format such as the impulse control disorder module of the SCID^53^ or the Minnesota Impulse Disorders Interview (MIDI;^54^, and inter-rater reliability assessment should be considered a limitation regarding internal validity. Fourth, the used GAD-7 focuses on generalized anxiety disorder and does not capture the full spectrum of anxiety disorders; however, clinical diagnoses allowed for a differentiated classification of anxiety disorders. Fifth, the clinical nature of the sample restricts generalizability to the broader population of individuals with CBSD who may not seek treatment.

### Clinical implications

In the clinical context, the findings of the present article underscore the importance of gender-sensitive assessment and treatment planning—particularly regarding product preferences and associated psychological motives, which may differ between women and men. To adequately address these differences, gender-specific aspects should be considered when selecting therapeutic modules, as evidence suggests that men and women may respond differently to psychotherapy^55^. Current German CBSD treatment guidelines recommend group therapy as the standard intervention^16^, although addressing gender-sensitive content in this format can be challenging. Therefore, investigating gender-specific aspects of CBSD is essential for tailoring treatment approaches and guiding the individual selection of therapeutic components. Such aspects could be addressed selectively, for instance, through individualized interventions or adapted group formats that account for gender-specific needs.

## Conclusion

In summary, women and men with CBSD appear largely similar in terms of clinical severity, psychiatric burden, and treatment engagement. The most robust gender differences were found in product preferences, suggesting that gender plays a more prominent role in the phenomenology of CBSD than in its overall clinical severity. These findings support a nuanced understanding of CBSD in which gender may shape behavioral expression rather than represent a primary risk factor. This also suggests that consumer policy and prevention strategies may benefit from considering gender-specific patterns of consumption. Future research should integrate gender more systematically into theoretical models and examine gender-related mechanisms in longitudinal and community-based samples. In addition, future studies should move beyond binary gender classifications and include gender-diverse and non-binary individuals to capture the full spectrum of gender-related aspects of CBSD.

## Data Availability

The data that support the findings of this study are not openly available due to reasons of sensitivity and are available from the corresponding author upon reasonable request.

## Declarations

### Animal studies

No animal studies were carried out by the authors for this article.
